# Associations between urban birth or childhood trauma and first-episode schizophrenia mediated by low IQ

**DOI:** 10.1038/s41537-022-00289-x

**Published:** 2022-10-29

**Authors:** Min Xie, Zhengyang Zhao, Minhan Dai, Yulu Wu, Yunqi Huang, Yunjia Liu, Yiguo Tang, Liling Xiao, Wei Wei, Guangya Zhang, Xiangdong Du, Chuanwei Li, Wanjun Guo, Xiaohong Ma, Wei Deng, Qiang Wang, Tao Li

**Affiliations:** 1 Mental Health Center, West China Hospital, Sichuan University, 610041 Chengdu, Sichuan China; 2 Department of Psychiatry, Suzhou Psychiatric Hospital, The Affiliated Guangji Hospital of Soochow University, Suzhou, China; 3 Affiliated Mental Health Centre & Hangzhou Seventh People’s Hospital, Zhejiang University School of Medicine, 310013 Hangzhou, Zhejiang China

**Keywords:** Schizophrenia, Neuroscience

## Abstract

Exposure to urban birth, childhood trauma, and lower Intelligence Quotient (IQ) were the most well-established risk factors for schizophrenia in developed countries. In developing countries, whether urban birth is a risk factor for schizophrenia and how these factors are related to one another remain unclear. This study aimed to investigate whether IQ mediates the relationship between urban birth or childhood trauma and first-episode schizophrenia (FES) in China. Birthplace, childhood trauma questionnaire (CTQ), and IQ were collected from 144 patients with FES and 256 healthy controls (HCs). Hierarchical logistic regression analysis was conducted to investigate the associations between birthplace, childhood trauma, IQ, and FES. Furthermore, mediation analysis was used to explore the mediation of IQ in the relationship between birthplace or childhood trauma and FES. After adjusting for age, sex and educational attainment, the final model identified urban birth (odds ratio (OR) = 3.15, 95% CI = 1.54, 6.44) and childhood trauma (OR = 2.79, 95% CI = 1.92, 4.06) were associated an elevated risk for FES. The 52.94% total effect of birthplace on the risk of FES could be offset by IQ (indirect effect/direct effect). The association between childhood trauma and FES could be partly explained by IQ (22.5%). In total, the mediation model explained 70.5% of the total variance in FES. Our study provides evidence that urban birth and childhood trauma are associated with an increased risk of FES. Furthermore, IQ mediates the relationship between urban birth or childhood trauma and FES.

## Introduction

It is estimated that about 70% of the world’s population will live in cities by 2050^1^. China has especially experienced an unprecedented rate of urbanization during the last decades. The proportion of the urban-dwelling population increased from 17.9% in 1978 to 52.6% in 2012^2^. Urbanization was associated with an increased risk of schizophrenia, and even there was a dose-response effect between them^3–5^. Several replications have been conducted, primarily in Europe^3,5–8^. However, one study included a nationally representative general population sample (*N* = 215,682) of adults residing in 42 low-income and middle-income countries and found that urbanization could increase the incidence of psychosis in Estonia, but opposite patterns were observed in Mali, Senegal, and the Philippines^9^. However, this study was conducted without considering different patterns of genetic, social, and biological exposures in different countries, so the conclusion might not be reliable^10^. Up to date, only one study found that urbanization was associated with an increased risk of schizophrenia (odds ratio (OR) = 1.44; 95% CI, 1.32 to 1.57) in China^11^. Therefore, it is an open question whether urbanization as a risk of schizophrenia differs between developed and developing countries.

Premorbid Intelligence Quotient (IQ) deficit, especially assessed in late adolescence, is a robust risk factor for the onset of schizophrenia^12,13^. Previous studies have confirmed that cognitive deficits are emergent in the early stage of neurodevelopment, including childhood and adolescence, before the onset of schizophrenia^14,15^. Kendler et al. found that the association between lower IQ and schizophrenia may be causal because genetic load for schizophrenia had a much stronger impact on the risk of illness for those with low IQ, rather than the result of shared familial risk factors^13^. Even though IQ is heritable and stable throughout adolescence to old age, it also has a high malleability affected greatly by normal environmental variation, including families, lifespan, and socioeconomic status (SES)^16,17^. In addition, environmental stressors, such as birth complications, substance abuse, urbanization, and childhood trauma, were associated with an increased risk of schizophrenia^18^. However, the potential impact of these environmental stressors on the cognition of schizophrenia has not been fully investigated in patients with schizophrenia.

Increased exposure to environmental stressors caused by urbanization, such as contamination of drinking water, air, and noise, impairs the development of brain structure and function, thus leading to cognition impairment^19–23^. However, more access to community resources (e.g., library, playgrounds, and community center), less financial difficulties (e.g., problems meeting basic needs such as food and housing), and less family adversity (e.g., lower father education and poor parental mental health) for children in an urban setting may lead to better neurocognitive development trajectory^24^. Therefore, it is unclear whether urban birth impedes or promotes cognitive development and whether IQ mediates these associations.

Childhood trauma, as adverse experiences in early childhood, including emotional abuse, physical abuse, sexual abuse, emotional neglect, and physical neglect, is a well-established risk factor for schizophrenia^25^. Many patients with schizophrenia spectrum disorders have experienced one or more childhood trauma events, and the most frequent subtype of trauma was emotional neglect^26^. A review of childhood trauma and neuroimaging studies showed that childhood trauma was associated with decreased gray matter in the prefrontal cortex, alterations of white matter integrity in several fiber bundles (the forceps major, inferior and superior longitudinal fasciculus, and the inferior fronto-occipital fasciculus), and abnormal functional connectivity in individuals with schizophrenia^27^. The structural and functional abnormalities of the brain associated with childhood trauma may be the neuropathological basis of cognitive dysfunction. One study found childhood maltreatment was associated with abnormal white matter integrity in posterior regions of the callosum and cognitive impairments in adolescents who experienced early life maltreatment^28^. Another study found that dysregulation of the hypothalamic-pituitary-adrenal (HPA) axis was associated with poor neurocognitive function^29^. In addition, a few studies found that childhood trauma was negatively associated with cognition in patients with schizophrenia^30,31^. Thus, we hypothesized that IQ may mediate between childhood trauma and schizophrenia.

However, the potential impact of these environmental stressors on the cognition of schizophrenia has not been fully investigated in patients with schizophrenia. In a developing country, the effect of IQ on the relationship between adverse environment exposure and schizophrenia remains unclear. This study aimed to (1) examine whether urban birth and childhood trauma are the risk factors for first-episode schizophrenia (FES) in China and (2) explore the mediating effect of IQ on the relationship between birthplace or childhood trauma and the development of FES.

## Methods

A total of 400 participants were enrolled in this study, including 144 patients with FES and 256 healthy controls (HCs). Patients with schizophrenia were recruited at the ward and out-patients clinic of the Psychiatric Department, West China Hospital, Sichuan University. HCs participants were recruited by poster and word-of-mouth advertisements in Chengdu and surrounding areas. All participants met the following inclusion criteria: (1) age range 16 to 45 years old; (2) Han Chinese; (3) right-handedness; (4) an IQ of 70 or above to exclude mental retardation. The participants (1) received a diagnosis of mental retardation, schizoaffective disorder, or other neurocognitive disorders; (2) suffered from severe head trauma or coma; (3) had an unstable medical condition; (4) were in the period of pregnancy or breastfeeding, were excluded. The diagnosis was assigned to DSM-IV diagnostic criteria according to the Structured Clinical Interview for DSM-IV (SCID) criteria, with SCID-P for patients and SCID-NP for HCs. All participants completed informed consent after a full explanation of the study procedures. Ethical approval for this study was granted by the Ethics Committee of the West China Hospital, Sichuan University.

### Clinical measurements

All subjects reported their birthplace (either rural or urban) according to the household register. Immigration status was based on the self-report of whether people moved to another place before 18. Childhood trauma was assessed by a childhood trauma questionnaire (CTQ), a 5-Likert score scale ranging from 25 to 125^32^. A positive and negative syndrome scale (PANSS) was used to evaluate the psychopathology of patients with FES. All assessments were performed by the researchers after unified training.

### Assessment of Wechsler Adult Intelligence Scale-Revised

The short version of the Wechsler Adult Intelligence Scale-Revised in China (WAIS-RC) includes 7 subtests, i.e., information, arithmetic, digital symbol coding, digital span, block design, picture completion, and similarities. The total Verbal IQ (VIQ) of scaled scores was obtained as 2(Information + Similarities) + Arithmetic + Digit Span; Total Performance IQ (PIQ) was calculated as 2(Picture Completion + Block Design) + Digit Symbol. The full scale of IQ (FSIQ) estimates was based on the sum scores of VIQ and PIQ. The estimated sums of scaled scores derived from these formulae were converted to IQ scores according to the standard procedure and age-corrected conversion tables in the WAIS-RC manual^33,34^.

### Analysis

T-test or analysis of variance (ANOVA) was carried out for group comparisons. Hierarchical logistic regression analysis was used to examine the associations between birthplace, CTQ, IQ and risk of schizophrenia. In the hierarchical analysis, the following variables were included: step 1 (age + sex + educational attainment); step 2 (variables retained from step 1 + birthplace); step 3 (variables retained from step 2 + CTQ); step 4 (variables retained from step 3 + FSIQ). All analyses above were based on version 15.0 of STATA (College Station, TX: StataCorp LLC.). Finally, mediation analysis was carried out to examine whether the mediating effects of IQ could explain the relationship between birthplace/childhood trauma and schizophrenia, with age, sex, body mass index (BMI) and educational attainment as covariates. Mediation analysis was based on version 8.4 of Mplus. Statistical significance was set at *P* < 0.05. Multiple comparisons were corrected by Bonferroni correction, and *P*_corrected_ < 0.05 was used as the significance threshold of correction.

## Results

**Fig. 1:**
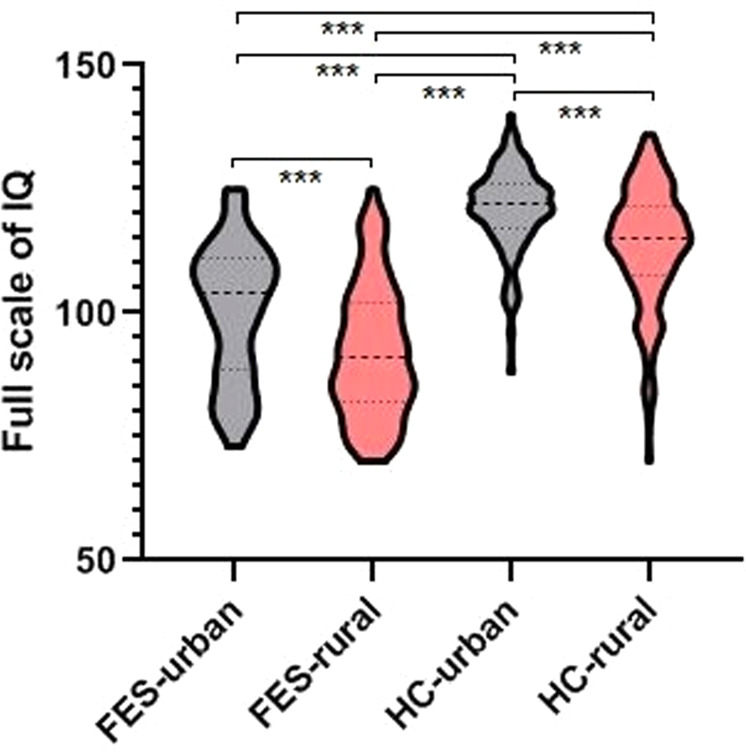
The comparison IQ in FES and HCs in different birthplaces. FES-urban: patients with FES born in urban areas, FES-rural: patients with FES born in rural areas, HC-urban: HCs born in urban areas, HC-rural: HCs born in rural areas.

Table 1 shows no difference in age, BMI, birthplace, and immigration status between FES and HCs (*P* > 0.05). Compared with HCs, education years and FSIQ of patients with FES were lower, and the percentage of males and CTQ of FES were higher (*P* < 0.05). As shown in Table 2, individuals with FES born in urban areas had fewer males, lower scores of PANSS, longer education years, and higher scores of FSIQ and GAF than FES born in rural areas (*P* < 0.05). In HCs, individuals born in urban areas had younger ages and higher FSIQ than individuals born in rural areas (*P* < 0.05). The FSIQ of patients with FES born in rural areas was lower than that of patients born in urban areas (all *P*_corrected_ < 0.001). FSIQ of patients with FES born in urban areas was lower than HCs born in urban and rural areas (all *P*_corrected_ < 0.001). FSIQ of HCs born in rural areas was lower than that of HCs born in urban areas (*P*_corrected_ < 0.001) (Fig. 1).

**Table 1:** Demographic and clinical information of FES and HCs.

Variable	All participants		Analysis	
	FES (*n* = 144)	HCs (*n* = 256)	Test statistic	*P*
Male sex, *n* (%)	64 (44.44)	82 (32.03)	χ^2^ = 6.127	0.013
Age (mean, SD)	23.50 (6.71)	23.71 (5.21)	*t* = 0.343	0.366
BMI (mean, SD)	20.72 (3.26)	20.82 (2.94)	*t* = 0.314	0.377
Education (years) (mean, SD)	12.38 (2.70)	15.75 (2.29)	*t* = 13.233	< 0.001
CTQ (mean, SD)	43.31 (13.40)	32.05 (7.07)	*t* = 10.923	< 0.001
Birthplace (urban, *n*, %)	58 (40.28)	113 (44.14)	χ^2^ = 0.562	0.454
Immigration (%)	25 (17.36)	65 (25.39)	χ^2^ = 3.408	0.065
FSIQ (mean, SD)	95.49 (14.10)	116.97 (11.32)	*t* = 13.676	< 0.001
DUP (months) (mean, SD)	15.16 (19.48)	–	–	–
Age of onset (mean, SD)	21.83 (6.20)	–	–	–
GAF (mean, SD)	47.82 (12.39)			
*PANSS (mean, SD)*				
Positive score	22.39 (6.18)	–	–	–
Negative score	21.82 (7.68)	–	–	–
General score	41.64 (10.70)	–	–	–
Total score	85.86 (20.10)	–	–	–

*FES* first-episode schizophrenia, *HCs* healthy controls, *BMI* body mass index, *CTQ* childhood trauma questionnaires, *FSIQ* the full scale of IQ, *DUP* duration of untreated psychosis, *GAF* global assessment function, *PANSS* Positive and Negative Syndrome Scale, *SD* standard deviation.

**Table 2:** Comparisons of demographic and clinical information between birthplace of rural and urban in FES and HCs.

Variable	FES		HCs	
	Rural	Urban	Rural	Urban
Male sex, *n* (%)	45 (52.33)	19 (32.76)*	47 (32.87)	35 (30.97)
Age (mean, SD)	22.35 (5.57)	23.14 (7.04)	24.50 (5.76)	22.71 (4.23)**
BMI (mean, SD)	20.47 (2.93)	21.11 (3.70)	20.62 (2.62)	21.09 (3.29)
Education (years) (mean, SD)	11.70 (2.60)	13.40 (2.53)***	15.75 (2.60)	15.76 (1.84)
CTQ (mean, SD)	43.61 (13.21)	42.86 (13.80)	32.01 (12.16)	32.09 (6.83)
FSIQ (mean, SD)	92.21 (13.40)	100.34 (13.82)***	113.71 (11.75)	121.10 (8.58)***
DUP (months) (mean, SD)	15.64 (18.86)	14.47 (20.49)	–	–
Age of onset (mean, SD)	21.46 (5.48)	22.43 (7.25)	–	–
GAF (mean, SD)	45.96 (11.91)	50.40 (12.68)*	–	–
*PANSS (mean, SD)*			–	–
Positive score	23.28 (5.98)	21.23 (6.30)*	–	–
Negative score	23.09 (7.49)	20.14 (7.66)*	–	–
General score	44.17 (10.02)	38.32 (10.75)***	–	–
Total score	90.55 (18.79)	79.68 (20.25)***	–	–

*FES* first-episode schizophrenia, *HCs* healthy controls, *BMI* body mass index, *CTQ* childhood trauma questionnaires, *FSIQ* the full scale of IQ, *DUP* duration of untreated psychosis, *GAF* global assessment function, *PANSS* Positive and Negative Syndrome Scale, *SD* standard deviation.

^*^P < 0.05; ^**^P < 0.01; ^***^P < 0.001.

The hierarchical logistic regression model (Table 3) showed that the covariates (age, sex, BMI, and educational attainment) accounted for 31.0% of the total variance in FES (step 1). Adding birthplace to step 1 (step 2) could explain an additional 4% of the variance. However, adding birthplace to step 2 could not improve the model fit (∆*R*^2^ = 0.004, χ^2^ = 2.390, *P* = 0.122). After accounting for the demographic and birthplace, childhood trauma contributed significantly to the fit of the model (∆R^2^ = 0.074, χ^2^ = 44.998, *P* < 0.001). Childhood trauma was associated with elevated risk of schizophrenia (OR = 2.76, 95% CI [1.98, 3.86], *P* < 0.001). The final model accounted for 49.0% of the total variability in FES, and the Omnibus test of the model was significant (∆*R*^2^ = 0.112, χ^2^ = 72.481, *P* < 0.001), indicating a good model fit. Furthermore, after adding FSIQ to step 3 (step 4), FSIQ was found to be a protective factor for schizophrenia (OR = 0.18, 95% CI [0.11, 0.28], *P* < 0.001). However, the effect of birthplace on FES was significant (OR = 3.15, 95% CI [1.54, 6.44], *P* = 0.002).

**Table 3:** Logistic regression models assessing the association of demographic variables, CTQ, FSIQ, urban birth and risk of schizophrenia.

	Step 1	Step 2	Step 3	Step 4
	OR (95% CI)	OR (95% CI)	OR (95% CI)	OR (95% CI)
Age	1.41 (1.05, 1.88)*	1.44 (1.08, 1.93)*	1.39 (1.02, 1.89)*	0.95 (0.67, 1.33)
Sex	1.46 (0.85, 2.53)	1.52 (0.88, 2.64)	1.30 (0.72, 2.35)	1.38 (0.69, 2.79)
Education (year)	0.18 (0.13, 0.27)***	0.17 (0.12, 0.26)***	0.21 (0.14, 0.32)***	0.45 (0.30, 0.69)***
BMI	0.90 (0.68, 1.18)	0.87 (0.66, 1.45)	0.91 (0.67, 1.22)	1.05 (0.73, 1.51)
Birthplace	–	1.51 (0.89, 2.56)	1.51 (0.85, 2.67)	3.15 (1.54, 6.44)**
CTQ	–	–	2.76 (1.98, 3.86)***	2.79 (1.92, 4.06)***
FSIQ	–	–	–	0.18 (0.11, 0.28)***
Model *R*^2^	0.310	0.314	0.388	0.490
Model *R*^2^_change_	–	∆*R*^2^ = 0.004	∆*R*^2^ = 0.074	∆*R*^2^ = 0.112
		χ^2^ = 2.39	χ^2^ = 44.998	χ^2^ = 72.481
		*P* = 0.122	*P* < 0.001	*P* < 0.001

*Step 1* effects of age, sex, and educational attainment, *Step 2* variables retained from step 1 and birthplace, *Step 3* variables retained from step 2 and CTQ, *Step 4* variables retained from step 3 and FSIQ. *CTQ* childhood trauma questionnaire, *IQ* intelligence quotient, *FSIQ* the full scale of IQ, *CI* confidence interval, *OR* odds ratio, *BMI* body mass index.

^*^P < 0.05, ^**^P < 0.01, ^***^P < 0.001.

**Fig. 2:**
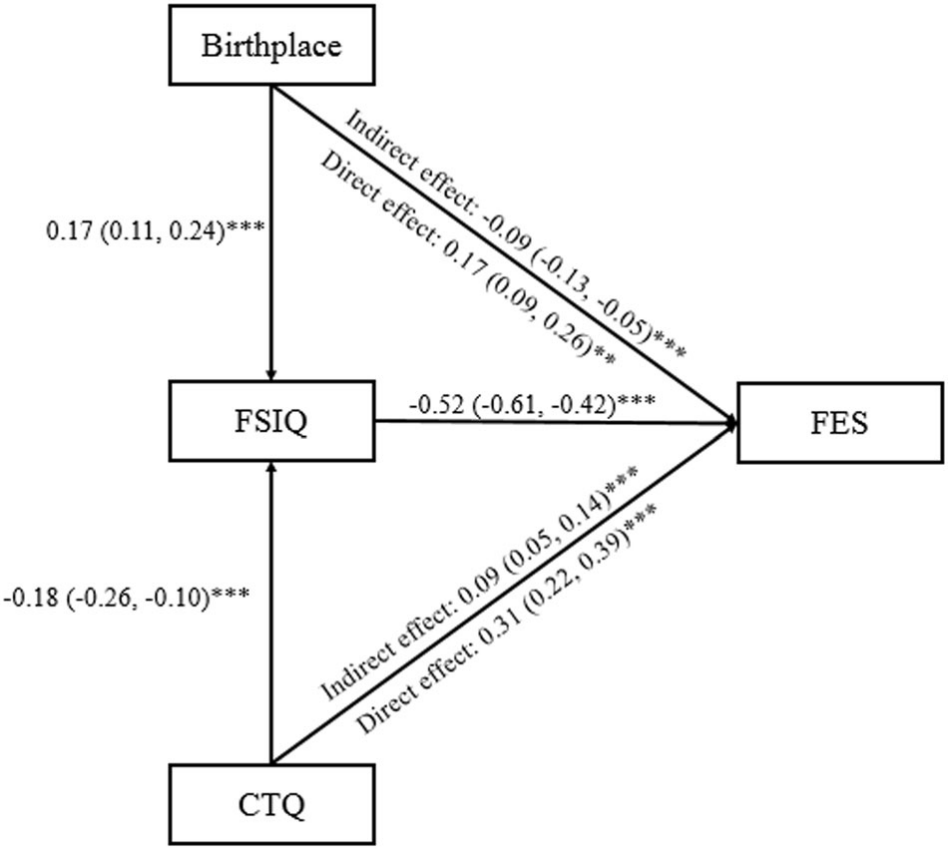
A mediation model of the association between birthplace, childhood trauma, and diagnosis through IQ. CTQ: childhood trauma questionnaire, FSIQ: the full scale of IQ, FES: first-episode schizophrenia. Age, sex, BMI, and educational attainment were adjusted in the mediation model. Estimates and 95% confidence interval in the parentheses are shown. ^**^P-value < 0.01, ^***^P-value < 0.001.

The mediation pathway model is shown in Fig. 2. Total effect of birthplace on diagnosis was not significant (β = 0.09, 95% CI [ − 0.01, 0.18], *P* = 0.120) after adjusting for age, sex, BMI, and educational attainment. The direct effect and indirect effect of birthplace on FES were both significant (β = 0.17, 95% CI [0.08, 0.25], *P* = 0.001; β = -0.09, 95% CI [-0.13, -0.05], *P* < 0.001). However, the signs of the indirect and direct effects were opposite. The total effects of birthplace on the risk of schizophrenia could be offset by about 52.94% (indirect effect/direct effect) via FSIQ. Total effect of childhood trauma on diagnosis was significant (β = 0.40, 95% CI [0.30, 0.49], *P* < 0.001), after adjusting for age, sex and educational attainment. The direct effect and indirect effect of childhood trauma on FES were significant (β = 0.31, 95% CI [0.22, 0.39], *P* < 0.001; β = 0.09, 95% CI [0.05, 0.14], *P* = 0.001). The association between childhood trauma and FES could be partly explained by FSIQ (proportion explained 22.5%). In total, the estimated model explained 70.5% of the total variance in FES (*R*^2^ = 0.705).

## Discussion

The primary purpose of this study was to evaluate the relationship between birthplace or childhood trauma, IQ and FES. The current study revealed the rural–urban difference in IQ of patients with schizophrenia. Furthermore, 51.46% of the risk of schizophrenia associated with urban birth was offset by IQ, and 23.12% of the risk of schizophrenia associated with childhood trauma could be attributable to indirect effects on IQ if these associations were causal.

Our results showed general cognitive differences between urban-born and rural-born individuals, consistent with previous studies^35,36^. Several potential possibilities have been proposed to explain the rural-urban gap in cognition. First, low socioeconomic status (SES) based on parental education, occupation, and income, was the most common phenomenon in rural areas^36,37^, especially in developing countries. SES is associated with declined scores on cognitive and language development and reduced cortical volume and thickness in children^38,39^. Second, lack of access to community sources (e.g., library, museum, and cultural activities center), lower quality of education (teachers with lower wages and levels of training), and reduced access to preschool education^24,36,40^. Third, significantly lower levels or lack of parenting sensitivity and stimulation in rural regions were associated with the poor performance of children^41,42^. In conclusion, the family environment, community, and school of childhood contribute to the difference in neurocognitive development between urban- and rural-born children.

Our findings suggest that urban birth is associated with an increased risk of schizophrenia after controlled IQ, which is consistent with the previous studies^3,11,43^. Previous studies revealed that built environment, environment pollution, social stress related to the urban milieu, and neighborhood characteristics were risk attributes of the urban environment^44,45^. These risk attributes could illuminate the relationship between urban exposure and schizophrenia. First, design meanings, triggers, symbols, etc., (high-rise buildings, narrow streets with no escape ways, less street planting and gardens, and billboards and flashing lights) of the built urban environment were associated with aberrant brain morphologies and reactivity patterns^46^. Furthermore, exposure to less green space in urban design was associated with a high incidence of psychological stress and schizophrenia^47,48^. Residential green space was crucial for developing intelligence and behavior in children living in urban areas^49^. Second, environmental pollution (e.g., air pollution, water pollution, and noise pollution) was common in urban areas. Exposure to air pollution, contamination of drinking, and chronic traffic noise in childhood were associated with long-term impairment of the HPA axis, brain structure, and function, which could be a potential neuropathological mechanism of schizophrenia^19,50–52^. Third, there was a huge income disparity in modern urban regions^53^. Individuals living in a poverty-stricken area were more likely to suffer from social stressors, crime victimization, and racial discrimination^54^. Indeed, city life-related social stress was associated with increased amygdala activity, and urban upbringing was related to increased pregenual anterior cingulate cortex activity, a critical region for stress regulation, amygdala activity, and neurocognitive processing^55^. Fourth, Kirkbride et al. found that neighborhood characteristics (neighborhood income inequality, absolute deprivation, population density, neighborhood ethnic composition effects, etc.) were associated with the risk of non-affective psychosis^8^. In addition, lack of physical activity and poor diets (low fruit and vegetable intake) were harmful to mental health^56,57^. Cumulative childhood and adolescence exposure to urban environments was associated with brain structure and function alterations. One study found a significant inverse correlation between urban upbringing and cortical thickness in the left dorsolateral PFC, bilateral medial PFC, and the temporal cortex, including the left superior temporal and left para-hippocampal cortex (*P* < 0.05, FWE-corrected)^58^. Urban exposure was a compound risk factor, including many environmental factors, which may require further study on the role of each element to make better intervention decisions.

Childhood trauma, independent of urban birth, is one of the risk factors for schizophrenia. The potential mechanisms may involve dysfunction of the HPA axis and alterations of neurobiological and epigenetic processes^59^. Both animal and human studies have confirmed that childhood trauma was related to long-term alterations of the HPA axis, including hyperdopaminergic state and augmented glucocorticoid release, which may affect specific brain regions (such as the hippocampus and the prefrontal cortex), leading to neurocognitive impairment^60,61^. Childhood trauma is associated with widespread alterations of brain structure and function, including decreased total cerebral gray matter (especially in the prefrontal cortex), decreased white matter integrity in the forceps major, inferior fronto-occipital fasciculus, inferior and superior longitudinal fasciculus, and functional connectivity of the amygdala and the anterior cingulate cortex^27^. In addition, epigenetics processes, such as DNA methylation and histone modifications, are important for cognition and symptoms in patients with schizophrenia^62,63^.

In our study, the association between birthplace and schizophrenia was mediated by IQ. A better understanding of cognition may better elucidate the underlying pathological mechanisms of environmental exposure and schizophrenia. IQ with high malleability was susceptible to environmental risk factors. Urban birth individuals were exposed to better education quality and more educational opportunities, as well as pollution, social stress, and bad living habits associated with urbanization. Furthermore, one study found that high IQ significantly reduces the effect of genetic susceptibility on the risk of schizophrenia^13^. Therefore, the total effect of urban birth on schizophrenia is not significant. In contrast, individuals exposed to deprivation (such as living in a poverty-stricken city area) were more likely to lack educational opportunities and suffer from more social stressors, crime victimization, and racial discrimination, which could impair cognitive development. Thus, urban birth was associated with an increased risk of schizophrenia in people with low IQ.

Additionally, our study found that low IQ partly contributed to the association between childhood trauma and schizophrenia. The adverse impact of childhood trauma on cognition may play a role in the developmental trajectory of schizophrenia, as low IQ is a well-established risk factor for schizophrenia^13^. This estimation strategy may account for some inconsistent evidence about the association between childhood trauma and schizophrenia in previous studies. Prior studies have focused solely on the main effect of childhood trauma on the increased risk of schizophrenia and have overlooked the possibility of the process via cognition linking them. A multicentric case-control study of first-episode psychosis found that childhood maltreatment was associated with cognition in psychosis and HCs^64^. However, the correlation is not the same as causation. A possible interpretation is that childhood trauma was associated with neurodevelopment, including alterations of brain structure and function^27,55,58^, which may be the neuropathological basis of cognitive deficits and the emergence of schizophrenia.

This study has several limitations. First, we did not collect cognitive data on the parents or siblings of the patients. Therefore, the familial liability both for IQ and schizophrenia was not considered. Second, the assessment of IQ used in this study was collected after the onset of illness. Although a previous study found that the IQ of patients with schizophrenia before onset was significantly lower than that of HCs, the effects of duration of untreated psychosis and illness of schizophrenia on IQ could not be excluded. Third, we could not examine the impact of cumulative urban exposure (urban exposure duration or quantified urbanization level) on schizophrenia. Fourth, although we controlled the effect of CTQ and IQ, our study could not prevent individual-level substance abuse, environmental pollutants, and low SES. Finally, the samples in this study were mainly from the Chengdu area of Sichuan province, a relatively developed region in China. The samples cannot well represent the complex characteristics and social and economic level of the Chinese population. Therefore, we cannot conclude that the findings of urbanization increasing the risk of schizophrenia in China are consistent with the results in developed countries.

## Conclusion

Urban birth and childhood trauma are associated with an increased risk of schizophrenia. Urban birth or childhood trauma may be involved in the onset of FES via a subsequent impact on cognition, which could possibly provide clues to the causal pathway of neurodevelopmental. This research could provide potential intervention targets, particularly in individuals born in urban areas or individuals who experienced childhood trauma, and strategies to promote cognitive development may give rise to protection from FES.

## Data Availability

The data that provide the findings of this study are available from the corresponding author upon reasonable request.

## References

[1] Acuto M, Parnell S, Seto KC (2018). Building a global urban science. Nat. Sustainability.

[2] Bai X, Shi P, Liu Y (2014). Society: realizing China’s urban dream. Nature.

[3] Vassos E, Pedersen CB, Murray RM, Collier DA, Lewis CM (2012). Meta-analysis of the association of urbanicity with schizophrenia. Schizophr. Bull..

[4] March, D. et al. Psychosis and place. *Epidemiol. Rev.* **30**, 84–100 (2008). 10.1093/epirev/mxn006 18669521

[5] Pedersen, C. & Mortensen, P. J. A. Evidence of a dose-response relationship between urbanicity during upbringing and schizophrenia risk. *Arch. Gen. Psychiatry.* **58**, 1039–1046 (2001). 10.1001/archpsyc.58.11.1039 11695950

[6] Marcelis M, Navarro-Mateu F, Murray R, Selten JP, Van Os J (1998). Urbanization and psychosis: a study of 1942–1978 birth cohorts in The Netherlands. Psychol. Med..

[7] Mortensen PB (1999). Effects of family history and place and season of birth on the risk of schizophrenia. N. Engl. J. Med..

[8] Kirkbride JB, Jones PB, Ullrich S, Coid JW (2014). Social deprivation, inequality, and the neighborhood-level incidence of psychotic syndromes in East London. Schizophr. Bull..

[9] DeVylder JE (2018). Association of urbanicity with psychosis in low- and middle-income countries. JAMA Psychiatry.

[10] Kirkbride JB, Keyes KM, Susser E (2018). City living and psychotic disorders-implications of global heterogeneity for theory development. JAMA Psychiatry.

[11] Luo Y, Pang L, Guo C, Zhang L, Zheng X (2021). Association of urbanicity with schizophrenia and related mortality in China: Association de l’urbanicité avec la schizophrénie et la mortalité qui y est reliée en Chine. Can. J. Psychiatry.

[12] Khandaker GM, Barnett JH, White IR, Jones PB (2011). A quantitative meta-analysis of population-based studies of premorbid intelligence and schizophrenia. Schizophr. Res..

[13] Kendler KS, Ohlsson H, Sundquist J, Sundquist K (2015). IQ and schizophrenia in a Swedish national sample: their causal relationship and the interaction of IQ with genetic risk. Am. J. Psychiatry.

[14] Bora E (2014). Developmental lag and course of cognitive deficits from the premorbid to postonset period in schizophrenia. Am. J. Psychiatry.

[15] Reichenberg A (2010). Static and dynamic cognitive deficits in childhood preceding adult schizophrenia: a 30-year study. Am. J. Psychiatry.

[16] van Ijzendoorn, M. H., Juffer, F. & Poelhuis, C. W. K. Adoption and cognitive development: a meta-analytic comparison of adopted and nonadopted children’s IQ and school performance. *Psychol. Bull.* **131**, 301–316 (2005). 10.1037/0033-2909.131.2.301 15740423

[17] Tucker-Drob EM, Briley DA (2014). Continuity of genetic and environmental influences on cognition across the life span: a meta-analysis of longitudinal twin and adoption studies. Psychol. Bull..

[18] Uher R, Zwicker A (2017). Etiology in psychiatry: embracing the reality of poly-gene-environmental causation of mental illness. World Psychiatry.

[19] Bondy, S. C. & Campbell, A. Water quality and brain function. *Int. J. Environ. Res. Public Health* **15**, 10.3390/ijerph15010002 (2017). 10.3390/ijerph15010002 PMC5800103 29267198

[20] Chew S (2020). Urban air particulate matter induces mitochondrial dysfunction in human olfactory mucosal cells. Particle Fibre Toxicol..

[21] Tregellas JR, Ellis J, Shatti S, Du YP, Rojas DC (2009). Increased hippocampal, thalamic, and prefrontal hemodynamic response to an urban noise stimulus in schizophrenia. Am. J. Psychiatry.

[22] Schilbach L, Eickhoff SB, Rotarska-Jagiela A, Fink GR, Vogeley K (2008). Minds at rest? Social cognition as the default mode of cognizing and its putative relationship to the “default system” of the brain. Consciousness Cognition.

[23] Hampson M, Driesen NR, Skudlarski P, Gore JC, Constable RT (2006). Brain connectivity related to working memory performance. J. Neuroscience.

[24] Robinson LR (2017). Differences in health care, family, and community factors associated with mental, behavioral, and developmental disorders among children aged 2-8 years in rural and urban areas—United States, 2011-2012. Morbidity Mortality Weekly Rep. Surveillance Summaries (Washington, D.C.: 2002).

[25] Matheson SL, Shepherd AM, Pinchbeck RM, Laurens KR, Carr VJ (2013). Childhood adversity in schizophrenia: a systematic meta-analysis. Psychol. Med..

[26] Larsson S (2013). High prevalence of childhood trauma in patients with schizophrenia spectrum and affective disorder. Comprehensive Psychiatry.

[27] Cancel A, Dallel S, Zine A, El-Hage W, Fakra E (2019). Understanding the link between childhood trauma and schizophrenia: A systematic review of neuroimaging studies. Neurosci. Biobehavioral Rev..

[28] Cunha PJ (2021). Callosal abnormalities, altered cortisol levels, and neurocognitive deficits associated with early maltreatment among adolescents: a voxel-based diffusion-tensor imaging study. Brain Behavior.

[29] Aas M (2011). Abnormal cortisol awakening response predicts worse cognitive function in patients with first-episode psychosis. Psychol. Med..

[30] Shannon C (2011). The association between childhood trauma and memory functioning in schizophrenia. Schizophr. Bull..

[31] Wells R (2020). The impact of childhood adversity on cognitive development in schizophrenia. Schizophr. Bull..

[32] Bernstein DP (2003). Development and validation of a brief screening version of the Childhood Trauma Questionnaire. Child Abuse Neglect.

[33] Ryan JJ, Weilage ME, Spaulding WD (1999). Accuracy of the seven subtest WAIS-R short form in chronic schizophrenia. Schizophr. Res..

[34] Gong Y-x (1983). Revision of Wechsler’s Adult Intelligence Scale in China. Acta Psychologica Sinica.

[35] Hermida MJ (2018). Risks for child cognitive development in rural contexts. Front. Psychol..

[36] Castro, J. & Rolleston, C. Gender equality and the empowerment of rural girls and young women: evidence from young lives (2015).

[37] Wang, X., Zhou, L. & Shang, X. Child poverty in rural china: multidimensional perspective. *Asian Social Work Policy Rev.* **9**, 10.1111/aswp.12050 (2015).

[38] Bradley RH, Corwyn RF (2002). Socioeconomic status and child development. Annu. Rev. Psychol..

[39] Tomasi D, Volkow ND (2021). Associations of family income with cognition and brain structure in USA children: prevention implications. Mol. Psychiatry.

[40] Green MJ, Pearce A, Parkes A, Robertson E, Katikireddi SV (2021). Pre-school childcare and inequalities in child development. SSM Popul. Health.

[41] Burchinal M, Vernon-Feagans L, Cox M, Key Family Life Project, I. (2008). Cumulative social risk, parenting, and infant development in rural low-income communities. Parent Sci. Pract..

[42] Mao, M., Zang, L. & Zhang, H. The effects of parental absence on children development: evidence from left-behind children in China. *Int. J. Environ. Res. Public Health* **17**, 10.3390/ijerph17186770 (2020). 10.3390/ijerph17186770 PMC7559575 32957472

[43] Allardyce J (2001). Comparison of the incidence of schizophrenia in rural Dumfries and Galloway and urban Camberwell. Br. J. Psychiatry: J. Mental Sci..

[44] Krabbendam L (2021). Understanding urbanicity: how interdisciplinary methods help to unravel the effects of the city on mental health. Psychol. Med..

[45] Abrahamyan Empson L (2020). Urbanicity: the need for new avenues to explore the link between urban living and psychosis. Early Intervention Psychiatry.

[46] Golembiewski JA (2016). The designed environment and how it affects brain morphology and mental health. Herd.

[47] Astell-Burt T, Feng X (2019). Association of urban green space with mental health and general health among adults in Australia. JAMA Network Open.

[48] Engemann K (2018). Childhood exposure to green space—a novel risk-decreasing mechanism for schizophrenia?. Schizophrenia Res..

[49] Bijnens EM, Derom C, Thiery E, Weyers S, Nawrot TS (2020). Residential green space and child intelligence and behavior across urban, suburban, and rural areas in Belgium: a longitudinal birth cohort study of twins. PLoS Med..

[50] Lubczyńska, M. J. et al. Air pollution exposure during pregnancy and childhood and brain morphology in preadolescents. *Environ. Res.* 110446, 10.1016/j.envres.2020.110446 (2020). 10.1016/j.envres.2020.110446 33221303

[51] Kuo SS, Pogue-Geile MF (2019). Variation in fourteen brain structure volumes in schizophrenia: a comprehensive meta-analysis of 246 studies. Neurosci. Biobehav. Rev..

[52] van Erp TGM (2018). Cortical brain abnormalities in 4474 individuals with schizophrenia and 5098 control subjects via the Enhancing Neuro Imaging Genetics Through Meta Analysis (ENIGMA) Consortium. Biol. Psychiatry.

[53] Massey DS (1996). The age of extremes: concentrated affluence and poverty in the twenty-first century. Demography.

[54] Bobo, L. Crime, urban poverty, and social science. *Du Bois Rev.: Social Sci. Res. Race* **6**, 10.1017/S1742058X0999021X (2009).

[55] Lederbogen F (2011). City living and urban upbringing affect neural social stress processing in humans. Nature.

[56] Biddle SJ, Asare M (2011). Physical activity and mental health in children and adolescents: a review of reviews. Br. J. Sports Med..

[57] O’Neil A (2014). Relationship between diet and mental health in children and adolescents: a systematic review. Am. J. Public Health.

[58] Besteher B, Gaser C, Spalthoff R, Nenadić I (2017). Associations between urban upbringing and cortical thickness and gyrification. J. Psychiatric Res..

[59] Popovic D (2019). Childhood trauma in schizophrenia: current findings and research perspectives. Front. Neurosci..

[60] Gomes, F., Zhu, X. & Grace, A. J. M. P. The pathophysiological impact of stress on the dopamine system is dependent on the state of the critical period of vulnerability. *Mol. Psychiatry* **25**, 3278–3291 (2019). 10.1038/s41380-019-0514-1 PMC7056584 31488866

[61] Van Voorhees E, Scarpa A (2004). The effects of child maltreatment on the hypothalamic-pituitary-adrenal axis. Trauma Violence Abuse.

[62] Misiak B (2015). Lower LINE-1 methylation in first-episode schizophrenia patients with the history of childhood trauma. Epigenomics.

[63] Bahari-Javan S (2017). HDAC1 links early life stress to schizophrenia-like phenotypes. Proc. Natl Acad. Sci. USA.

[64] Sideli L (2022). Childhood maltreatment, educational attainment, and IQ: findings from a multicentric case-control study of first-episode psychosis (EU-GEI). Schizophr. Bull..

